# Feature Extraction and Similarity of Movement Detection during Sleep, Based on Higher Order Spectra and Entropy of the Actigraphy Signal: Results of the Hispanic Community Health Study/Study of Latinos

**DOI:** 10.3390/s18124310

**Published:** 2018-12-06

**Authors:** Miguel Enrique Iglesias Martínez, Juan M. García-Gomez, Carlos Sáez, Pedro Fernández de Córdoba, J. Alberto Conejero

**Affiliations:** 1 Departamento de Telecomunicaciones, Universidad de Pinar del Río, Pinar del Río, Cuba, Martí #270, CP: 20100; Instituto Universitario de Matemática Pura y Aplicada, Universitat Politècnica de València (UPV), Camino de Vera s/n, 46022 Valencia, España; migueliglesias2010@gmail.com; 2 Biomedical Data Science Lab (BDSLab), Instituto Universitario de Tecnologías de la Información y Comunicaciones (ITACA), Universitat Politècnica de València (UPV), Camino de Vera s/n, 46022 Valencia, España; carsaesi@ibime.upv.es; 3 Instituto Universitario de Matemática Pura y Aplicada, Universitat Politècnica de València (UPV), Camino de Vera s/n, 46022 Valencia, España; pfernandez@mat.upv.es (P.F.d.C.); aconejero@upv.es (J.A.C.)

**Keywords:** actigraphy, bispectrum, entropy, feature extraction

## Abstract

The aim of this work was to develop a new unsupervised exploratory method of characterizing feature extraction and detecting similarity of movement during sleep through actigraphy signals. We here propose some algorithms, based on signal bispectrum and bispectral entropy, to determine the unique features of independent actigraphy signals. Experiments were carried out on 20 randomly chosen actigraphy samples of the Hispanic Community Health Study/Study of Latinos (HCHS/SOL) database, with no information other than their aperiodicity. The Pearson correlation coefficient matrix and the histogram correlation matrix were computed to study the similarity of movements during sleep. The results obtained allowed us to explore the connections between certain sleep actigraphy patterns and certain pathologies.

## 1. Introduction

Actigraphy is now being increasingly used to explore sleep patterns in sleep laboratories. Its main advantages include its easy setup, its low cost, and the fact that prolonged records can be obtained over time, permitting patient activity in ambulatory conditions without interfering with their daily routines. It is considered to be a valuable tool for controlling and monitoring circadian alterations and insomnia, as well as avoiding false positives in the assessment of daytime sleepiness tests, such as the multiple sleep latency test, and the wakefulness maintenance test [1,2,3,4,5].

Many recent studies have validated the practice of actigraphy, for example, in [6] several wrist-worn sleep assessments, actigraphy devices were compared. A relationship has been found between sleep disorders and their effects on certain conditions, such as hypertension and obesity [7], and it is now even possible to analyze sleep depth by actigraphy signals [8].

A review of the current state of higher-order statistics (HOS) and their use in biosignal analysis can be found in [9]. As most of the biomedical signals are non-linear, non-stationary, and non-Gaussian in nature, iHOS (Higher Order Statistics) analysis is preferable to second-order correlations and power spectra [9]. On this issue, several studies, such as [10] have been published on the screening of pediatric sleep apnea–hypopnea syndrome, and the automated classification of glaucoma stages in [11].

Concerning the detection of similarity of movements, in [12,13] although classification patterns were obtained from sleep/awake states according to the characteristics of the actigraphy signal, they were not based on higher order spectra. In fact, the common approach is to analyze individual actigraphy records over several days, so that the studies cited above were not focused on the analysis of the activity signal as a random process that is dependent on the movement of a certain part of the body.

The present work is based on the bispectral analysis of actigraphy signals and their relationship with bispectral entropy. The increase of movements as a form of feature extraction measurement, and the detection of similarities of movements during sleep are shown as features to be considered. The results obtained indicate the potential of this approach for the study of sleep disorders, and their connection with other conditions. The work is organized as follows: Materials and Methods are described in Section 2, the results are given in Section 3, the Discussion in Section 4, and the Conclusions and future work are outlined in Section 5.

## 2. Materials and Methods

### 2.1. Data Acquisition

The experiments were carried out on 20 samples of actigraphy signals obtained from the Hispanic Community Health Study/Study of Latinos (HCHS/SOL) Database [14,15,16,17] chosen at random, through the use of the “randi” Matlab function. The Sueño Ancillary Study recruited 2252 HCHS/SOL participants to wear wrist-worn actigraphy devices (Actiwatch Spectrum, Philips Respironics, Royal Philips, Netherlands,) between 2010 and 2013. The participants were instructed to wear the watch for a week. Records were scored by a trained technician of the Boston Sleep Reading Center [17].

### 2.2. Methods

Actigraphy signals have a random nature that can be visualized in terms of uniformity in the bispectrum. This uniformity depends on the non-impulsive characteristics of the signal, which are reflected in the spectrum as frequency peaks. Since the bispectrum is a function that presents unique characteristics for each signal in terms of frequency and phase it can easily be seen in a graph. This led us to explore an entire methodology based on calculating the bispectrum and the bispectral entropy, which would be able to detect similar characteristics in movement patterns during sleep. Twenty cases of actigraphy signals were analyzed to extract their characteristics, which were then used to determine similarities and differences among the signals.

The activity signals were first normalized to 1, and then segmented to determine the subjects’ daily activity record. The bispectrum of the total sample of the activity signal recorded was seven days. The experiments were conducted on two age groups between 18 and 44 years old, and 45 and 64 years old.

### 2.3. Theoretical Foundations: Bispectrum

Let ${\left\{x\left(n\right)\right\}}_{n},\;n=0,\pm 1,\pm 2,\ldots$ be a stationary random vector, and let us also suppose that we can compute its higher order moments [18,19], where:

(1) $$m_{k}^{x}\left(\tau_{1},\tau_{2},\ldots ,\tau_{k-1}\right)=E\left(x\left(n\right)\cdot x\left(n+\tau_{1}\right)\ldots x\left(n+\tau_{k-1}\right)\right)$$

represents the moment of order k of that vector. This moment only depends on the different time slots $\tau_{1},\ldots ,\;\tau_{k-1}$ where $\tau_{i}=0,\pm 1,\ldots$ for all i. The cumulants are similar to the moments, but the difference is that the moments of a random process are derived from the characteristic function of the random variable, while the cumulant generating function is defined as the logarithm of the characteristic function of that random variable. The k-th order cumulant of a stationary random process ${\left\{x\left(n\right)\right\}}_{n}$ can be written as [20]:

(2) $$c_{k}^{x}\left(\tau_{1},\tau_{2},\ldots ,\tau_{k-1}\right)=m_{k}^{x}\left(\tau_{1},\tau_{2},\ldots ,\tau_{k-1}\right)-m_{k}^{G}\left(\tau_{1},\tau_{2},\ldots ,\tau_{k-1}\right),$$

where $m_{k}^{G}\left(\tau_{1},\tau_{2},\ldots ,\tau_{k-1}\right)$ is the k-th order moment of a process with an equivalent Gaussian distribution that presents the same mean value and autocorrelation function as the vector ${\left\{x\left(n\right)\right\}}_{n}$.

It is evident from (2) that a process following a Gaussian distribution has null cumulants for orders greater than 2, since $m_{k}^{x}\left(\tau_{1},\tau_{2},\ldots ,\tau_{k-1}\right)=m_{k}^{G}\left(\tau_{1},\tau_{2},\ldots ,\tau_{k-1}\right),$ and so that $c_{k}^{x}\left(\tau_{1},\tau_{2},\ldots ,\tau_{k-1}\right)=0$ [20,21].

In practice, we estimate cumulants and polyspectra from a finite amount of data ${\left\{x\left(n\right)\right\}}_{n=0}^{N-1}$. These estimates are also random and are characterized by their bias and variance [22]. Let ${\left\{x\left(n\right)\right\}}_{n}$. denote a zero mean stationary process; we assume that all relevant statistics exist, and that they have finite values. The third order cumulant sample estimate is given by [21]:

(3) $$C_{3}\left(\tau_{1},\tau_{2}\right)=\frac{1}{N}\sum_{n=N_{1}}^{N_{2}}x\left(n\right)\cdot x\left(n+\tau_{1}\right)\cdot x\left(n+\tau_{2}\right)$$

where $N_{1}$ yand $N_{2}$ are chosen such that the sums only involve *x*(*n*) for n=0,…, N−1, N being the number of samples in the cumulant region. Likewise, the bispectrum estimation is defined as the Fourier Transform of the third-order cumulant sequence [22]:

(4) $$B_{x}^{N}\left(f_{1},f_{2}\right)=\sum_{\tau_{1}=-N-1}^{N-1}\sum_{\tau_{2}=-N-1}^{N-1}C_{3}\left(\tau_{1},\tau_{2}\right)\cdot e^{-2\pi f_{1}\tau_{1}}\cdot e^{-2\pi f_{2}\tau_{2}}=\frac{1}{N^{2}}X^{*}\left(f_{1}+f_{2}\right)\cdot X\left(f_{1}\right)\cdot X\left(f_{2}\right)$$

where $f_{1}$ and $f_{2}$ are the spectral frequency vectors of the sequence ${\left\{x\left(n\right)\right\}}_{n=0}^{N-1}$, and $X\left(f_{i}\right)$, *i* = 1, 2, is its Fourier Transform.

### 2.4. Bispectral Entropy Analysis

Entropy provides a measure for quantifying the information content of a random variable in terms of the minimum number of bits per symbol that are required to encode the variable. It is an indicator of the amount of randomness or uncertainty of a discrete random process [23]. Consider a random variable Z with M states $z_{1},z_{2},\ldots z_{M}$, and state probabilities $p_{1},p_{2},\ldots p_{M}$, that is, $P\left(Z=z_{i}\right)=p_{i}$, the entropy of Z is defined as:

(5) $$H\left(Z\right)=-\sum_{i=1}^{M}p_{i}\log_{2}\left(p_{i}\right)$$

The entropy of a discrete-valued random variable attains a maximum value for a uniformly distributed variable. In order to extend this notion from the spatial to the frequency domain, we introduce bispectral entropy as a way of measuring the uniformity of the spectrum [21]. The bispectral entropy is defined as:

(6) $$E_{bx}^{N}\left(f_{1},f_{2}\right)=-\overset{N-1}{\underset{\tau_{1}=-N-1}{\sum }}\overset{N-1}{\underset{\tau_{2}=-N-1}{\sum }}P_{x}^{N}\left(f_{1},f_{2}\right)\cdot \log_{2}P_{x}^{N}\left(f_{1},f_{2}\right)$$

where the energy probability is computed in terms of the bispectrum estimation:

(7) $$P_{x}^{N}\left(f_{1},f_{2}\right)=\frac{B_{x}^{N}\left(f_{1},f_{2}\right)}{\sum_{\tau_{1}=-N-1}^{N-1}\sum_{\tau_{2}=-N-1}^{N-1}B_{x}^{N}\left(f_{1},f_{2}\right)}$$

## 3. Results

The actigraphy signals that measured the movements of individuals while sleeping were analyzed. These movements have an intrinsically random nature, since they can occur with non-specific probabilities and durations. This can be checked by analyzing the frequency spectrum of the activity signal and comparing it with a noise pattern. The probabilistic distribution function of the spectral pattern depends on the nature and uniformity of the movements, which may follow a normal distribution or another, such as a uniform distribution, depending on the random nature of the process.

### 3.1. Application of the Bispectrum to the Actigraphy Signal

**Figure 1:**
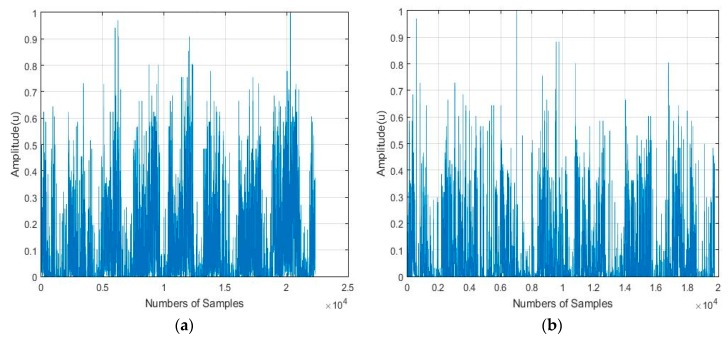
Ilustration of: (**a**,**b**) Examples of the frequency spectrum of two actigraphy signals obtained from their respective one-dimensional Fourier transforms.

A spectral analysis based on the one-dimensional Fourier transform is not recommended for the detection of traits in a random signal, such as the actigraphy signal. For these, this analysis only provides information relative to the magnitude-frequency or phase-frequency distribution. In other words, what is visualized in the spectrum is noise, which in our case, is in fact the useful information from which certain characteristics and features have to be extracted. The frequency spectrum of two actigraphy signals is shown in Figure 1, where it can be seen that the one-dimensional Fourier Transform is not able to identify the discriminant features in this type of signal.

**Figure 2:**
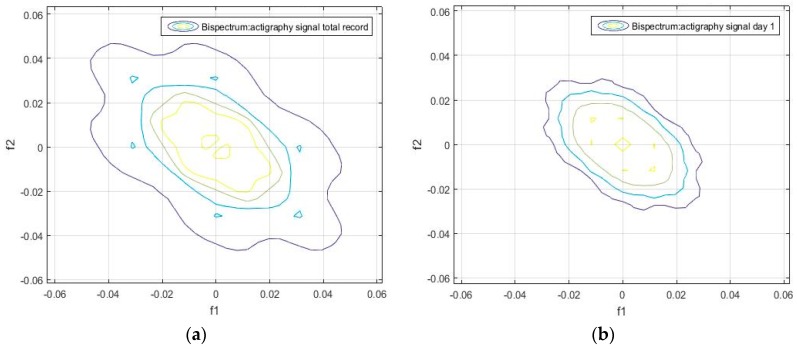
(**a**) Bispectrum of the activity record over seven days, and (**b**) bispectrum of the activity record on day 1 of the actigraphy data sample hchs-sol-sueno-00163225.

**Figure 3:**
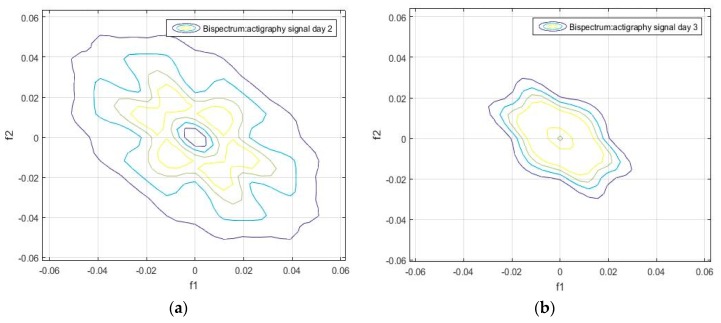
(**a**) Bispectrum of the activity record on day 2, and of (**b**) bispectrum of the activity record on day 3 of the actigraphy data sample hchs-sol-sueno-00163225.

Unlike the one-dimensional frequency spectrum, the bispectrum of an activity signal can provide information on the spatial distribution of the amplitude, and on the frequency components (see Equation (4)). This information can be represented in a matrix that can be used to obtain the particular identification features of each signal. The bispectrum of the actigraphy signal was simulated in MatLab, using the Higher Order Spectra Analysis toolbox. Figure 2 and Figure 3 show the contours of the bispectrum surface of the actigraphy signal, where f1 and f2 are the normalized spectral frequency vectors generated from the calculation of the bidimensional Fourier Transform. 

**Figure 4:**
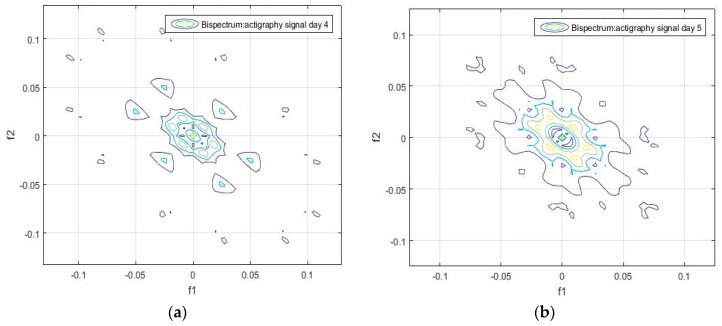
(**a**) Bispectrum of the activity record on day 4, and (**b**) bispectrum of the activity record on day 5 of the actigraphy data sample hchs-sol-sueno-00163225.

**Figure 5:**
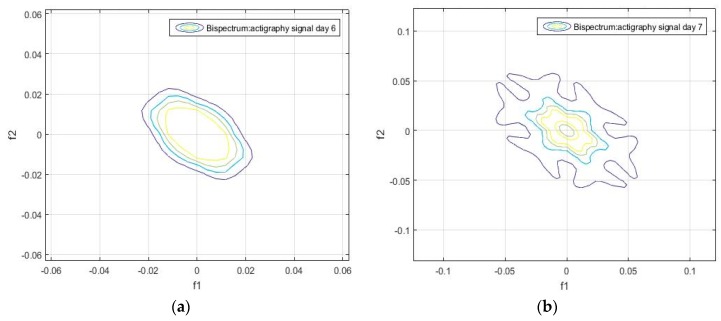
(**a**) Bispectrum of the activity record on day 6, and (**b**) bispectrum of the activity record on day 7 of the actigraphy data sample hchs-sol-sueno-00163225.

We found that the bispectrum can indicate variables that measure specific characteristics of the movement during sleep, based on the uniformity of the activity data and the disorder of the sample. Here, a greater frequency disorder at a bispectral level may imply an excess of movement during the analyzed period, which can even be an identifying feature of sleep, and be linked to patients. For the sake of completeness, we can see in Figure 2, Figure 3, Figure 4 and Figure 5 that the bispectrum is a unique variable for each actigraphy signal.

It can also be seen that the daily bispectrum registrations are all different from each other, showing that all these registers form an identification pattern, which we have named the bispectral pattern of the activity signal.

**Figure 6:**
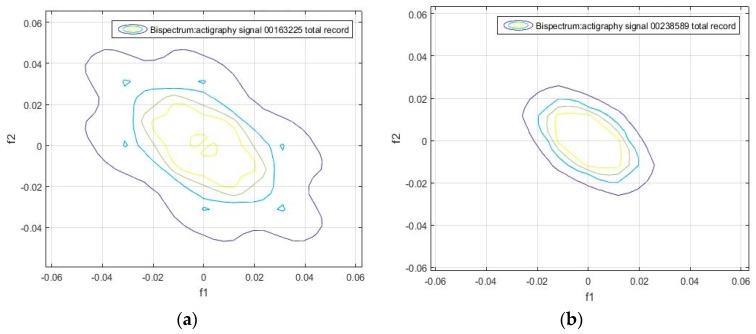
Bispectrum obtained from the 7-day activity record of the samples (**a**) hchs-sol-sueno-00163225and (**b**) hchs-sol-sueno-00238589.

**Figure 7:**
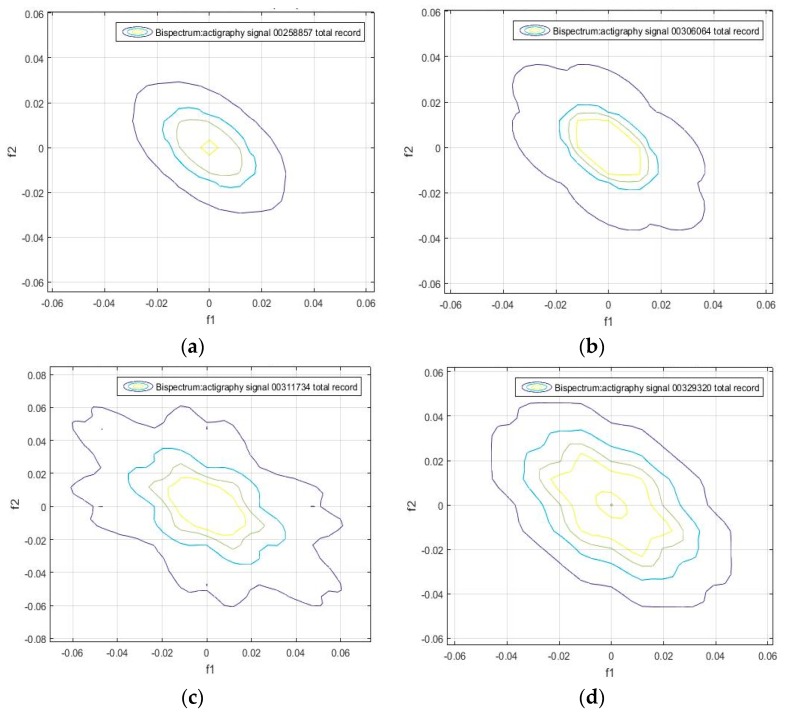
Bispectrum obtained from the 7-day activity record of the samples (**a**) hchs-sol-sueno-00258857, (**b**) hchs-sol-sueno-00306064, (**c**) hchs-sol-sueno-00311734, and (**d**) hchs-sol-sueno-00329320.

**Figure 8:**
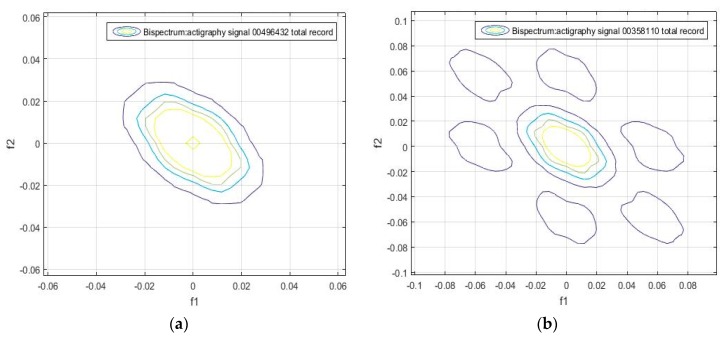

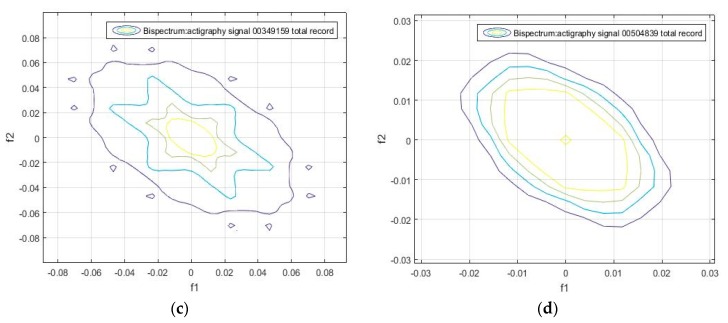
Bispectrum obtained from the 7-day activity record of the samples (**a**) hchs-sol-sueno-00349159 (**b**) hchs-sol-sueno-00358110 (**c**) hchs-sol-sueno-00496432 (**d**) hchs-sol-sueno-00504839.

A bispectrum analysis was performed on 20 different activity signal records. We tried to identify each one with a specific spectral sleep pattern per day, and to find a possible relationship between an individual’s movement patterns during sleep. The results obtained are shown in Figure 6, Figure 7 and Figure 8, which give the bispectrum of the actigraphy signal for the first 10 of the 20 analyzed actigraphy signals from the HCHS/SOL database.

It can be seen that there are unique identifiable characteristic features that can be used to obtain patterns of movement during sleep. For instance, Figure 5a, Figure 6b, Figure 7a and Figure 8d have similar contours. This means individuals can be divided into groups according to the similarity of their sleep patterns.

To further illustrate these results, we correlated the bispectrum of the seven days of signals by computing the Pearson correlation coefficients for every pair of samples to find similarities between the two signals. The results are given in the correlation matrix R in Table 1. For example, $R_{1-2}$ is the Pearson correlation coefficient between the bispectrum of samples 1 and 2 from hchs-sol-sueno-00163225 and hchs-sol-sueno-00238589.

**Figure 9:**
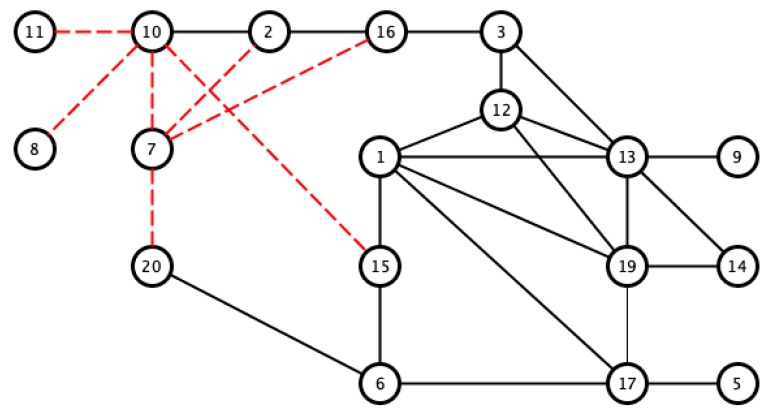
Visualization of pairs with Pearson correlation coefficients greater than 0.97 (black line) and lower than 0.8 (red dashed line).

In order to determine subgroups in the set of samples, and to identify the pairs of signals that give correlation values closest to 1, we selected the pairs with correlation values of greater than 0.97. This was done to satisfy the hypothesis of the similarity of the sleep movement patterns of two signals, since there must be as few differences as possible, and therefore, also minimal differences in their bispectral patterns. The results of similar pairs are shown in black in Figure 9, in which the values with the lowest correlation are indicated with red dashed lines to show different activity patterns. For this latter case, we considered values of below 0.8. Although these values are relatively high in comparison with other applications, we have considered its use for the search of dissimilar sleep patterns.

**Figure 10:**
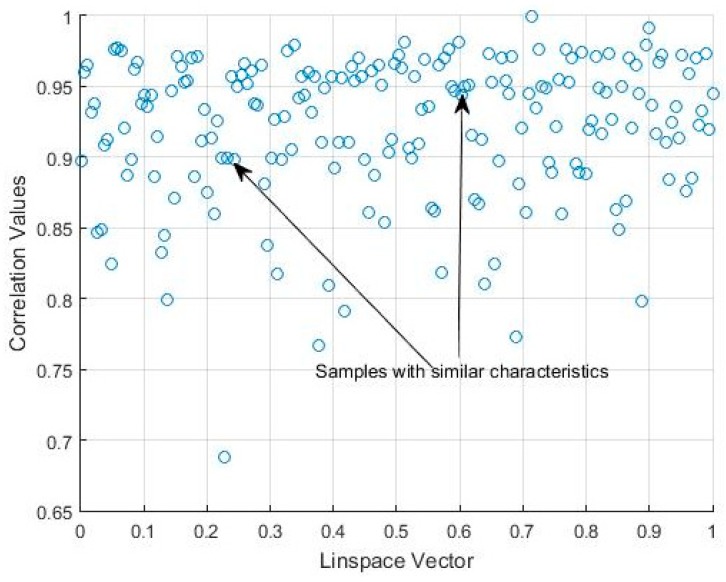
Scatter plot of the correlation matrix shown in Table 1.

The correlation values given in Table 1 and Figure 9 show that there may be a similarity in sleep movement patterns. In Table 1, the maximum distance value is 0.3122 and the minimum is $10^{-6}$, the mean is 0.0538, and the statistical mode (the most frequent value in an array) is 0.001. Figure 10 gives a comparative measurement of the values in Table 1 by rearranging the columns of the matrix into a vector, and considering it as a time series, in which the x-coordinate is the position in the vector and the y-coordinate, the corresponding value of the coefficient. In this arrangement, the groups indicate almost repetitive terms that represent signals with similar characteristics.

In order to better distinguish the differences and similarities between the sleep signals, we performed another analysis using the bispectral entropy as the method of characterizing the disorder/uniformity of the processed signals.

### 3.2. Application of Bispectral Entropy as a Measure of Actigraphy Disorder

The experiment was based on a similarity analysis, analogous to that of the bispectrum. We calculated the bispectral entropy of each activity sample for the whole period of seven days, to obtain a measure of the degree of uniformity of the sleep movement pattern, taking the degree of randomness of the activity signal into account. We considered the maximum value of the bispectral entropy as a way of describing the degree of uniformity of a random process.

**Figure 11:**
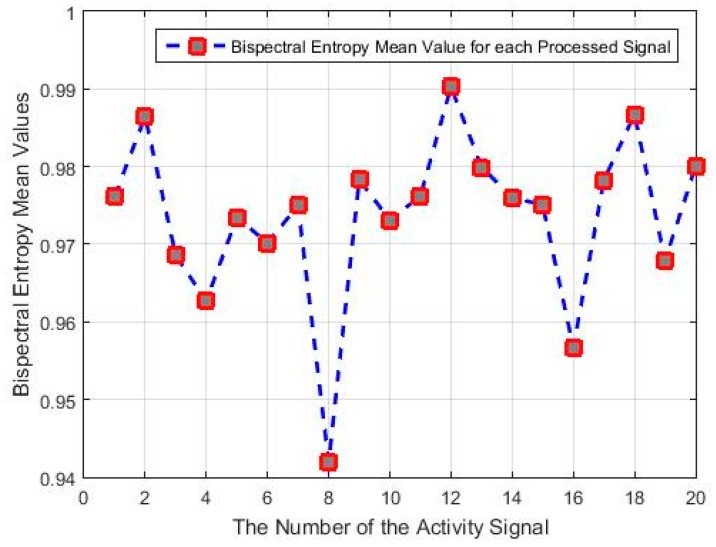
Mean bispectral entropy values of the 20 actigraphy signals considered.

The bispectral entropy of the signals was computed in a minimum window of eight samples, to represent the temporal displacement index of the signals. The results obtained are shown in Figure 11, together with the mean value of the bispectral entropy of each actigraphy signal.

It can be seen that signals 8 and 16 have the lowest bispectral entropy values, due to the non-uniformity of the bispectrum frequency distribution. This can also be identified in some of the previous graphs; for instance, in Figure 8b, the high-frequency components are characterized by the outer points (in blue), and the disconnected regions are the lowest frequency values.

In Figure 11 there are also samples with similar values of bispectral entropy of between 0.98 and 0.99, which indicates that they may be related to the hypothesis that activity samples with a similar correlation at the bispectral level may have the same level of uniformity of their value distributions. The opposite is also true with the minimum values of bispectral entropy, shown in Figure 11, as are those of samples 8, 10, 7, and 16, and other visible relationships, whose correlation values are under 0.8 in Table 2, and in Figure 11 are related to different uniformity patterns.

Given the analogy of the activity signal with the random process, the maximum entropy value would mean a greater uniformity of movement in the subject in the time interval studied, i.e., a high uniformity in the randomness of the movements. Conversely, occasional movements would be associated with impulsive noise, which has a non-uniform randomness, and thus, it would be associated with minimum entropy.

**Figure 12:**
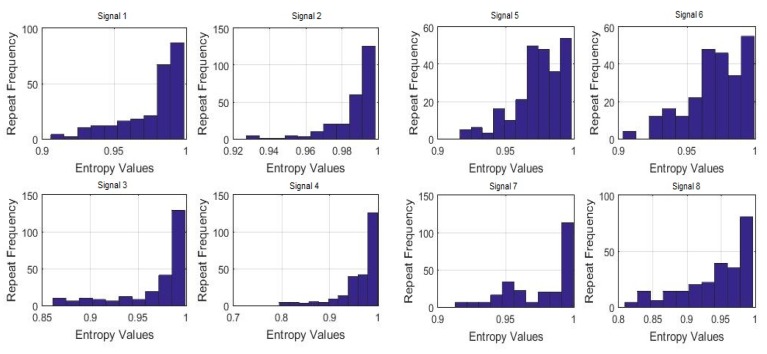
Histograms of the 7-day bispectral entropy of each activity signal (Signals 1 to 8, processed samples).

**Figure 13:**
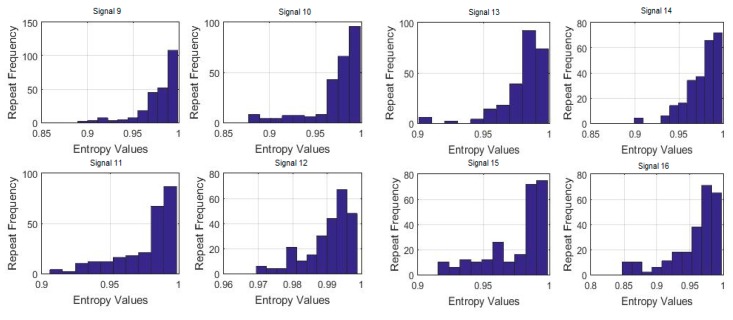

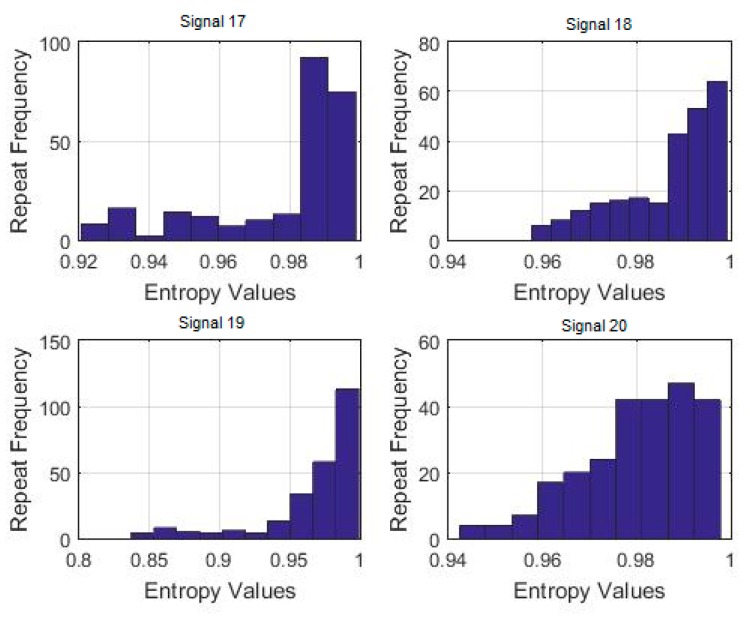
Histograms of the 7-day bispectral entropy of each activity signal (Signals 9 to 20, processed samples).

To also visualize the frequency of the maximum uniformity of sleep movements, histograms were made of the 7-day bispectral entropy of each activity signal. The frequencies of the entropy values for each processed sample are shown in Figure 12 and Figure 13. These histograms provide information on the number of repetitions of the entropy values in each sample, i.e., the number of times the value in the data vector is repeated.

Although none of the histograms is repeated in Figure 12 and Figure 13, some of them show certain similarities that could indicate similar sleep patterns. To verify this, the histograms were correlated to each other, with the criteria for the entropy values as well as for the data repetition frequency. The results are shown below in Table 2.

Table 2 contains the results based on the histogram of the bispectral entropy of the activity signals to provide a criterion for the similarity of the data, based on the uniformity of the bispectrum. This table can be interpreted similarly to Table 1, which was based on the algorithm that describes the matrix correlation in Figure 9.

**Figure 14:**
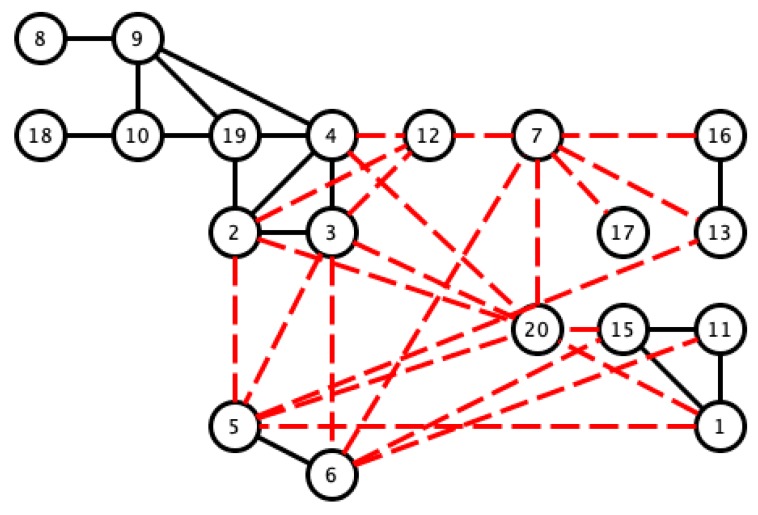
Visualization of pairs with Pearson correlation coefficients greater than 0.97 (black line) and lower than 0.7 (red dashed line).

According to the previous analysis, the upper threshold was 0.97, and the lower threshold was a little lower than previously found. We considered 0.7 to distinguish between the similarities and clear differences among the signals (see Figure 14).

**Figure 15:**
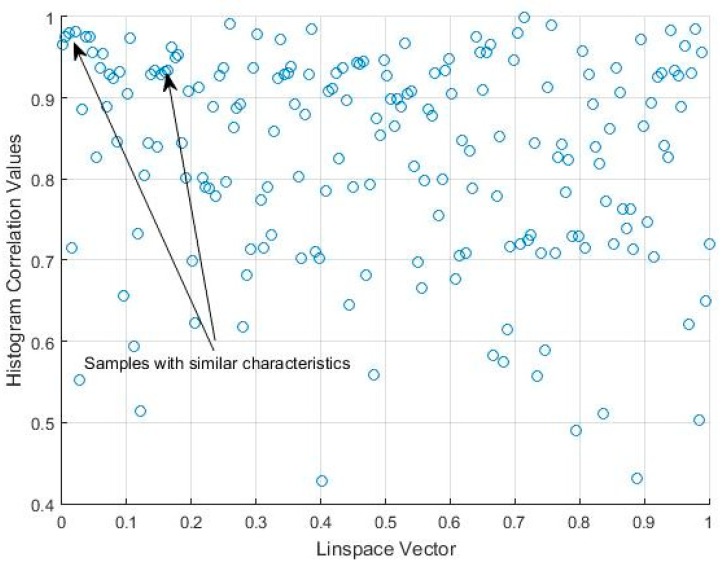
Scatter plot for the correlation matrix shown in Table 2.

It can thus be seen that several histograms are highly correlated, which indicates that this activity signal presents a high level of data uniformity, i.e., bispectral entropies with similar values, and also a high correlation value in terms of the bispectrum comparison. The dispersion graph of the correlation values obtained from Table 2 is shown in Figure 15. The data with similar values are seen to be grouped. The maximum value of the distance matrix is 0.6715, and the minimum is $10^{-5}$. The mean value of the distance matrix was 0.1407, and the statistical mode was $10^{-5}$, which indicates data groups with similar characteristics associated with the same type of movement, as can be seen in Figure 15.

## 4. Discussion

In order to associate the results with clinical diagnoses, several variables were taken from the HCHS/SOL database as the clinical characteristics of the 20 actigraphy samples. First, we considered the following variables:

CDCR_SUENO: self-report of cerebrovascular disease & carotid revascularization.
CHD_SELF_SUENO: combination of self-reports of coronary revascularization or heart attack.
DIABETES_SELF_SUENO: indicates a self-report of diabetes.
DIABETES _SUENO: indicates diabetes.
DM_AWARE_SUENO: describes the awareness of diabetes.
Hypertension_SUENO: indicates hypertension status.
STROKE_SUENO: checks for a self-report of stroke history.
STROKE_TIA_SUENO: checks for medical history of stroke, mini-stroke or TIA (transient ischemic attack).

These variables are of the 0/1 type, i.e., ‘0’ for a negative response and ‘1’ for a positive. Their values for the 20 individuals whose actigraphy signals were processed can be found in Table 3.

To relate the clinical characteristics of the patients with the obtained results, the correlation was first used, which is a measure of the similarity of data. We show these results, although the obtained correlations are weak, in part, for the limited number of signals used, and for the limitations of the information content embedded in the used signals database.

**Figure 16:**
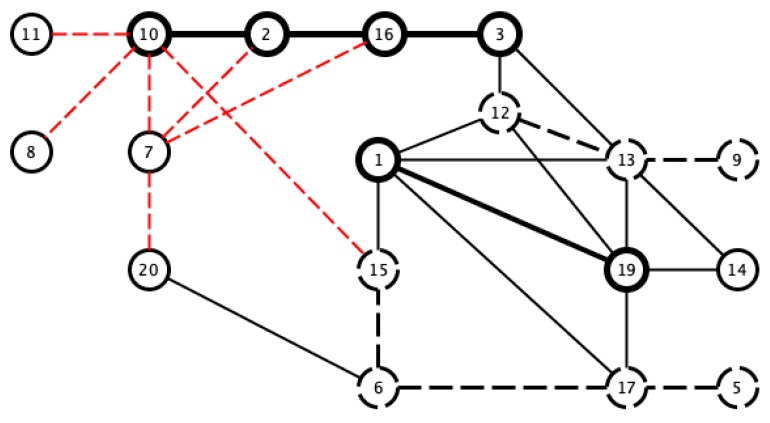
Pairs of bispectrum signals correlated with a coefficient that is greater than 0.97 (black lines) or lower than 0.7 (red dashed line). The thick black line indicates pairs that share a hypertension diagnosis, while the dashed black line indicates pairs in which neither has hypertension.

We opted to consider the HYPERTENSION_SUENO variable to study relationships within the actigraphy signals, since its value varies in several samples. First, we saw that 47.62% of the pairs whose bispectrum correlates with a value greater than 0.97 share the same clinical diagnosis. However, in Figure 9, it can be seen that the pairs with the same positive or negative diagnosis tend to cluster, which indicates a stronger hidden relationship that cannot be obtained by simply correlating the bispectrum of the signals (see Figure 16).

**Figure 17:**
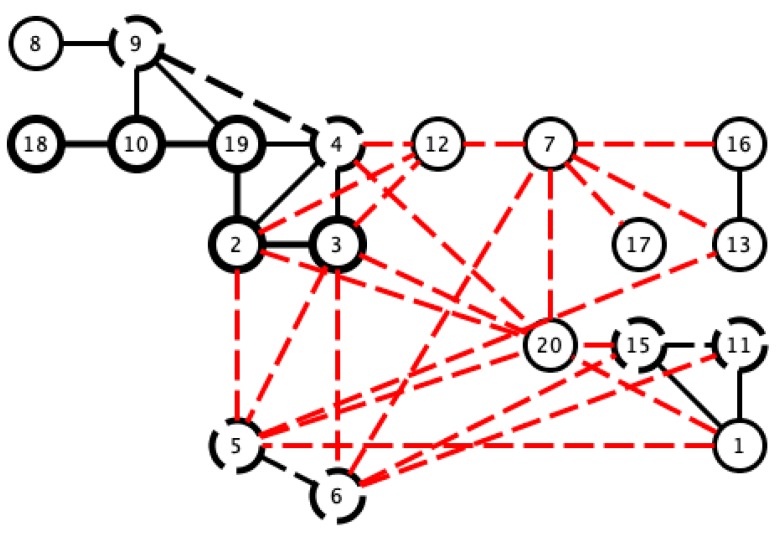
Pairs of bispectral entropy histograms correlated with a coefficient greater than 0.97 (black lines), and lower than 0.7 (red dashed line). The thick black line indicates pairs with a shared hypertension diagnosis, while the dashed black line indicates pairs in which neither has hypertension.

A similar effect was found in the comparison of the bispectral entropy histograms. Only 41.17% of the pairs correlated with a coefficient of 0.97 or higher present the same hypertension diagnoses. However, in the pairs with the same diagnosis in Figure 14 those sharing the hypertension diagnosis are seen to be connected (see Figure 17).

Although, the results shown in Figure 16 and Figure 17 are not conclusive, they do suggest a further in-depth study of the characteristics of bispectrum signals that can contribute most to these similarities. It is also worth mentioning that the limited number of cases considered in this study advise a more systematic study of larger database samples.

## 5. Conclusions

This paper has shown that the application of higher-order statistical analysis to actigraphy signals can contribute to determining the traits and patterns of movement during sleep. These criteria can be based on part of the spatial information provided by the bispectrum and the bispectral entropy, both of which can help us to determine effective criteria for measuring the uniformity of data randomness.

The actigraphy signal experiments suggest the possible application of these criteria for the extraction and comparison of patterns of sleep movements. This would have a potential use in medicine, since similar pathologies may have similar associated movement patterns.

In future work we propose to use high-order statistical techniques, as for instance in [23]. We also want to experiment with data from chest actigraphy or other actigraphy signal measures, to corroborate the potential use of sleep actigraphy signals for purposes of diagnosis.

Our next step will be to increase the number of cases analyzed to cover the entire HCHS/SOL database, and also to experiment with other clinical characteristics in patients and pathologies associated with specific sleep disorders or brain-associated diseases.

## Figures and Tables

**Table 1:** Correlation matrix obtained from the analysis of the bispectrum comparison of the 7-day activity signal for the 20 Hispanic Community Health Study/Study of Latinos (HCHS/SOL) database samples analyzed.

0.898	0.944	0.934	0.965	0.957	0.899	0.899	0.947	0.825	0.966	0.976	0.971	0.950	0.979	0.911	0.972	0.970	0.973	0.945
0.961	0.935	0.875	0.881	0.767	0.860	0.957	0.981	0.823	0.935	0.953	0.949	0.869	0.991	0.884	0.876	0.923	0.919	-
0.965	0.944	0.914	0.837	0.911	0.961	0.909	0.892	0.970	0.976	0.970	0.917	0.970	0.937	0.925	0.959	0.933	-	-
0.931	0.886	0.860	0.899	0.949	0.887	0.886	0.949	0.954	0.950	0.895	0.945	0.920	0.916	0.936	0.885	-	-	-
0.938	0.914	0.926	0.927	0.809	0.962	0.969	0.951	0.945	0.949	0.890	0.973	0.965	0.967	0.913	-	-	-	-
0.847	0.832	0.899	0.817	0.948	0.951	0.936	0.915	0.971	0.896	0.974	0.926	0.945	0.972	-	-	-	-	-
0.849	0.844	0.688	0.889	0.892	0.854	0.864	0.870	0.773	0.889	0.888	0.863	0.799	-	-	-	-	-	-
0.908	0.799	0.887	0.929	0.911	0.904	0.862	0.867	0.881	0.921	0.920	0.849	-	-	-	-	-	-	-
0.913	0.891	0.957	0.976	0.956	0.913	0.965	0.912	0.921	0.955	0.926	-	-	-	-	-	-	-	-
0.739	0.871	0.898	0.905	0.791	0.966	0.819	0.810	0.861	0.860	-	-	-	-	-	-	-	-	-
0.946	0.924	0.892	0.964	0.841	0.960	0.960	0.941	0.922	-	-	-	-	-	-	-	-	-	-
0.977	0.964	0.958	0.942	0.964	0.963	0.976	0.953	-	-	-	-	-	-	-	-	-	-	-
0.975	0.953	0.966	0.957	0.954	0.981	0.949	-	-	-	-	-	-	-	-	-	-	-	-
0.921	0.954	0.952	0.944	0.970	0.906	-	-	-	-	-	-	-	-	-	-	-	-	-
0.888	0.970	0.961	0.960	0.957	-	-	-	-	-	-	-	-	-	-	-	-	-	-
0.899	0.886	0.937	0.931	-	-	-	-	-	-	-	-	-	-	-	-	-	-	-
0.962	0.971	0.937	-	-	-	-	-	-	-	-	-	-	-	-	-	-	-	-
0.967	0.912	-	-	-	-	-	-	-	-	-	-	-	-	-	-	-	-	-
0.938	-	-	-	-	-	-	-	-	-	-	-	-	-	-	-	-	-	-

**Table 2:** Correlation matrix obtained from the analysis of the bispectral entropy histograms of the 20 analyzed samples from the HCHS/SOL database.

0.966	0.906	0.908	0.681	0.702	0.791	0.889	0.934	0.957	1.000	0.828	0.928	0.938	0.972	0.931	0.927	0.931	0.957	0.720
0.976	0.973	0.699	0.714	0.880	0.944	0.967	0.948	0.966	0.725	0.842	0.893	0.906	0.865	0.841	0.889	0.985	0.650	-
0.979	0.593	0.623	0.937	0.930	0.943	0.906	0.906	0.583	0.731	0.783	0.840	0.763	0.748	0.827	0.964	0.504	-	-
0.715	0.732	0.913	0.979	0.985	0.945	0.908	0.677	0.780	0.845	0.824	0.818	0.740	0.894	0.983	0.621	-	-	-
0.981	0.514	0.802	0.774	0.711	0.681	0.816	0.706	0.853	0.558	0.730	0.511	0.762	0.703	0.934	-	-	-	-
0.553	0.805	0.790	0.715	0.702	0.793	0.697	0.848	0.575	0.708	0.490	0.773	0.713	0.926	-	-	-	-	-
0.886	0.845	0.788	0.791	0.429	0.559	0.665	0.709	0.615	0.589	0.729	0.863	0.430	-	-	-	-	-	-
0.975	0.929	0.889	0.731	0.786	0.874	0.798	0.834	0.717	0.913	0.958	0.720	-	-	-	-	-	-	-
0.976	0.934	0.779	0.859	0.908	0.855	0.887	0.789	0.947	0.989	0.714	-	-	-	-	-	-	-	-
0.957	0.840	0.927	0.925	0.912	0.947	0.878	0.975	0.980	0.708	-	-	-	-	-	-	-	-	-
0.828	0.928	0.938	0.972	0.931	0.927	0.931	0.957	0.720	-	-	-	-	-	-	-	-	-	-
0.937	0.932	0.796	0.928	0.825	0.898	0.756	0.910	-	-	-	-	-	-	-	-	-	-	-
0.955	0.934	0.992	0.931	0.937	0.865	0.800	-	-	-	-	-	-	-	-	-	-	-	-
0.889	0.962	0.863	0.939	0.897	0.899	-	-	-	-	-	-	-	-	-	-	-	-	-
0.929	0.950	0.887	0.892	0.646	-	-	-	-	-	-	-	-	-	-	-	-	-	-
0.924	0.954	0.892	0.802	-	-	-	-	-	-	-	-	-	-	-	-	-	-	-
0.846	0.845	0.618	-	-	-	-	-	-	-	-	-	-	-	-	-	-	-	-
0.932	0.801	-	-	-	-	-	-	-	-	-	-	-	-	-	-	-	-	-
0.657	-	-	-	-	-	-	-	-	-	-	-	-	-	-	-	-	-	-

**Table 3:** Clinical characteristics of each individual analyzed for each actigraphy sample.

Samples	CDCR_SUENO	CHD_SELF_SUENO	DIABETES_SELF_SUENO	DIABETES_SUENO	DM_AWARE_SUENO	HYPERTENSION_SUENO	STROKE_SUENO	STROKE_TIA_SUENO
1	0	0	0	0	0	1	0	0
2	1	1	1	1	1	1	0	0
3	0	0	0	0	0	1	0	0
4	0	0	0	0	0	0	0	0
5	0	0	0	0	0	0	0	0
6	0	0	0	0	0	0	0	0
7	0	0	0	1	0	1	0	0
8	0	0	0	0	0	1	0	0
9	0	0	0	0	0	0	0	0
10	0	0	0	0	0	1	0	0
11	0	0	0	0	0	0	0	0
12	0	0	0	0	0	0	0	0
13	0	0	0	0	0	0	0	0
14	0	0	0	0	0	0	0	0
15	0	0	0	0	0	0	0	0
16	0	0	0	0	0	1	0	0
17	0	0	0	1	0	0	0	0
18	0	1	0	0	0	1	0	0
19	0	0	1	1	1	1	0	0
20	0	0	0	0	0	1	0	0
